# Supplementary material for: Nonmagnetic band gap at the Dirac point of the magnetic topological insulator (Bi1−xMnx)2Se3
Source: Nat Commun. 2016 Feb 19;7:10559. doi: 10.1038/ncomms10559 (PMC4762886; doi:10.1038/ncomms10559)
Supplement: Supplementary Information — Supplementary Figures 1-21, Supplementary Tables 1-2, Supplementary Notes 1-9 and Supplementary References [file ncomms10559-s1.pdf]

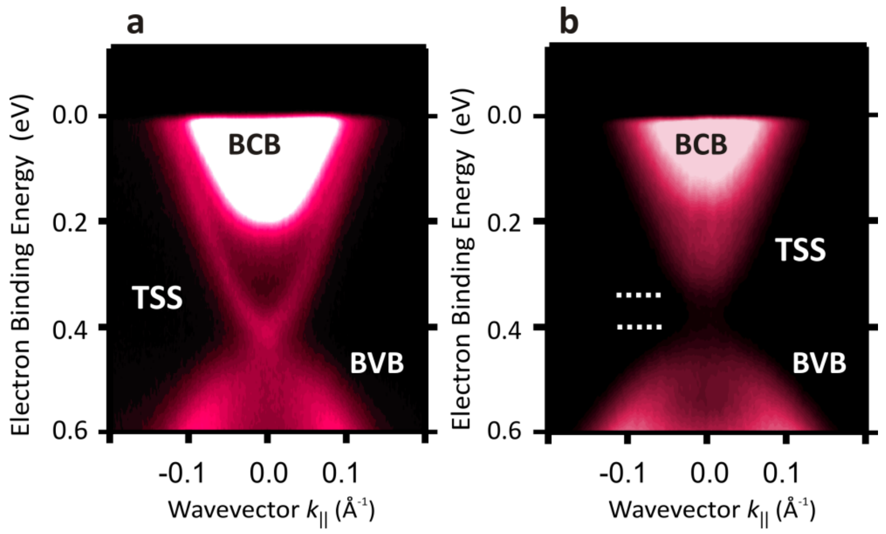

Supplementary Figure 1: **Probing the bulk-conduction band at low photon energy.** In contrast to Fig. 1 of the main text, the bulk-conduction band (BCB) is probed at 21 eV. (a, b) Energy-momentum ARPES dispersions of (a) undoped Bi_2Se_3 and (b) 8% Mn-doped Bi_2Se_3. The upward shift of the BCB in (b) is due to the p-type doping and not to a change in the bulk band gap or even a reversal of the bulk band inversion (see also Supplementary Fig. 15). The topological surface state (TSS) appears with weaker photoemission intensity which makes it difficult to observe when it approaches the BCB for 8% Mn in (b). The signal from the bulk-valence band (BVB) is also observed at higher binding energies. The TSS is better visible at 50 eV in Fig. 1 of the main text and in Supplementary Fig. 3. The horizontal white-dashed lines in (b) highlight the opening of the surface gap.

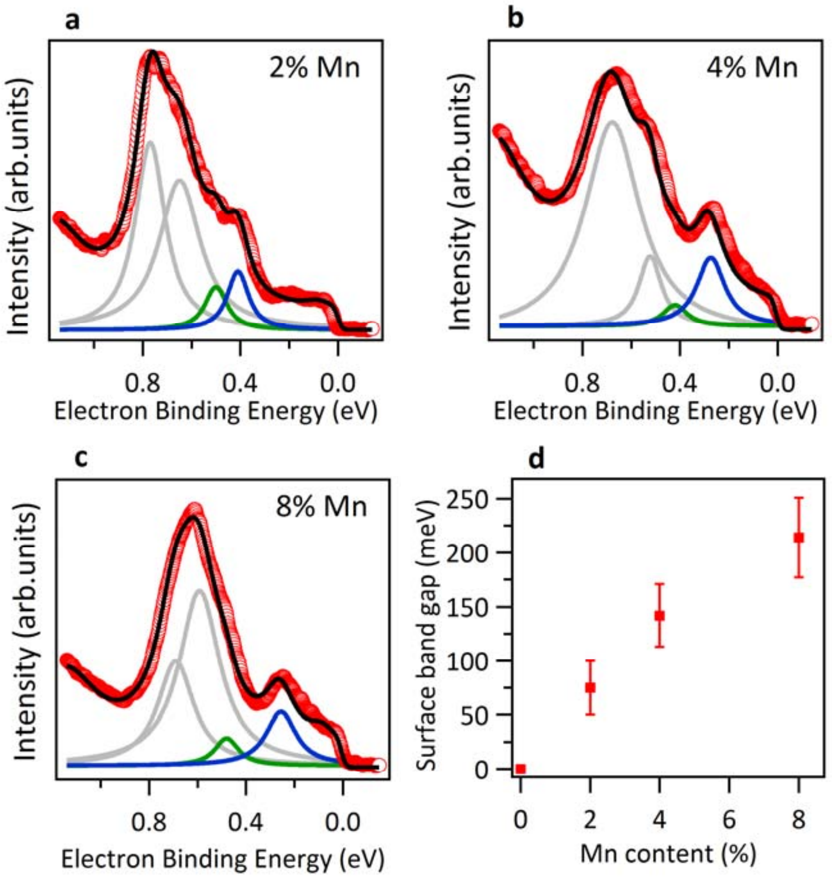

Supplementary Figure 2: **Surface band gap for different Mn concentrations.** (a-c) The experimental energy-distribution curves (EDCs) at zero momentum ($k_{||}$=0, red circles), obtained from the ARPES spectra acquired at 50 eV photon energy and 12 K temperature, are fitted (solid black lines) considering a Shirley-like background [1]. The fitting procedure is explained in detail in Supplementary Note 1. The fit results for (a) 2%, (b) 4% and (c) 8% Mn-doped Bi_2Se_3 films are shown. (d) Size of the surface band gap obtained from the fits in (a-c). The surface band gap is determined from the energy separation between the fitted Lorentzian peaks shown in blue (green) color, which are located at the energy minimum (maximum) of the upper (lower) half of the Dirac cone. The Lorentzian peaks shown in gray color are contributions from the bulk-valence bands. Error bars in (d) correspond to the uncertainty of determining the energy position of the band dispersions. The error bars are estimated from the standard deviations of the peak positions over several fitting cycles.

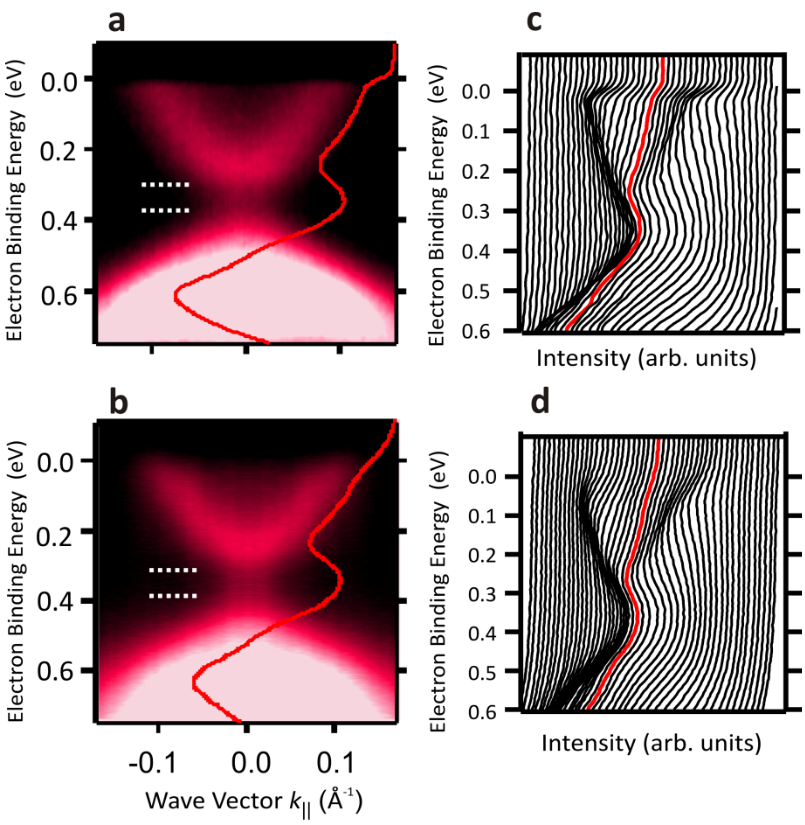

Supplementary Figure 3: **Temperature dependence of the surface band gap.** (a, b) Data as in Fig. 2 of the main text for 8% Mn-doped Bi_2Se_3 and (c, d) in a different representation as energy-distribution curves (EDCs), showing the absence of a temperature dependence of the surface band gap. Measurements were obtained at a temperature of (a, c) 12 K and (b, d) and 300 K. The red solid lines in (a-d) emphasize the EDCs in normal emission ($k_{||}$=0). The horizontal white-dashed lines in (a, b) highlight the surface band gap.

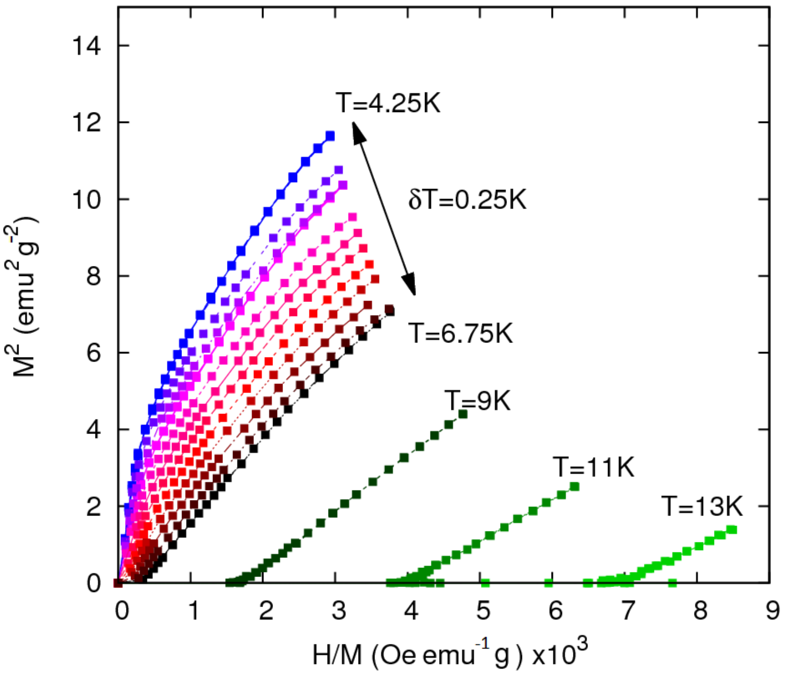

Supplementary Figure 4: **Conventional mass-normalized Arrott plot.** In Fig. 3c of the main text, we showed a modified Arrot plot according to a 3D Heisenberg ferromagnet [2, 3] normalized to the mass of the sample with the exponents β= 0.348 and γ = 1.41, from which we deduce a T_C of 5.5 K. Here we show for the same data a conventional mass-normalized Arrott plot. A similar presentation was used previously [4] for the determination of the ferromagnetic Curie temperature in $(Bi_{1-x}Mn_x)_2Te_3$. From the present plot, we deduce a by 2 K higher T_C.

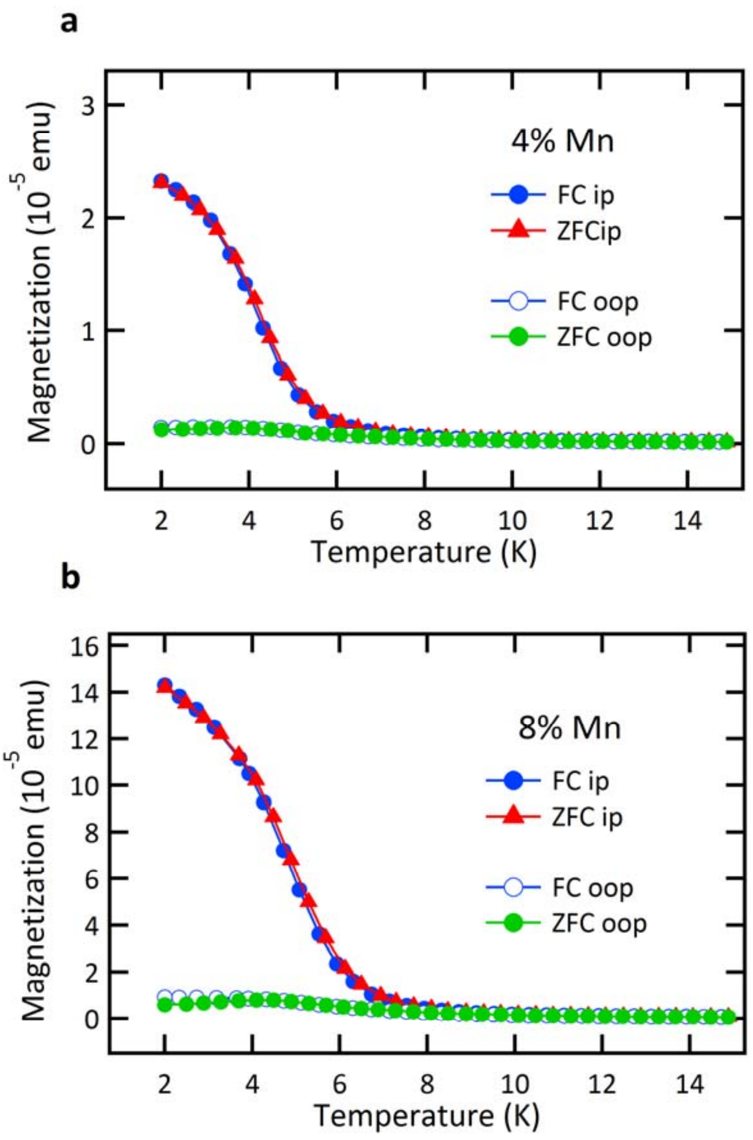

Supplementary Figure 5: **Temperature-dependence of the bulk magnetization.** Comparison between field-cooling (FC) and zero-field-cooling (ZFC) measurements obtained using SQUID magnetometry for (a) 4% and (b) 8% Mn doping. The temperature dependence of both the in-plane (ip) and out-of-plane (oop) components of the magnetization is shown. The FC measurements are performed under an applied magnetic field of 10 mT parallel and perpendicular to the surface. The ZFC and FC results are very similar for each composition, and compare well to the surface-sensitive XMCD measurements shown in the main text (see Supplementary Note 2 for details).

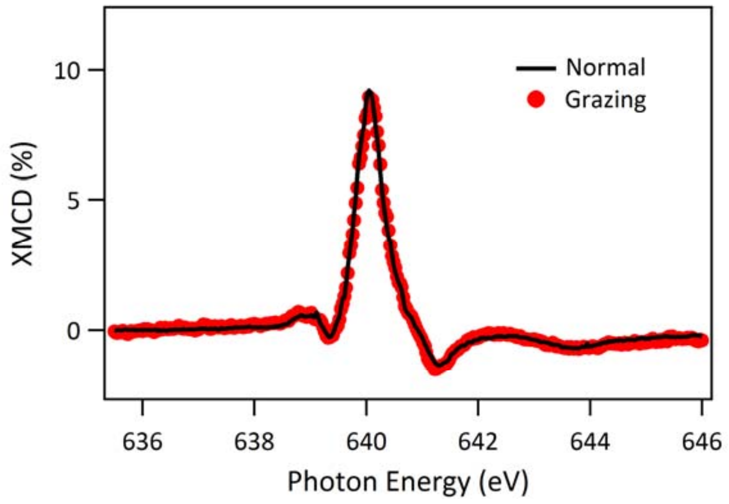

Supplementary Figure 6: **Surface-sensitive XMCD measurements for 8% Mn-doped Bi_2Se_3.** The spectra were obtained at the Mn L_3-edge and a temperature of 20 K under an applied magnetic field of -2T parallel (red circles) and perpendicular to the surface (black solid line). The x-ray beam impinged the sample in normal incidence (black solid line) and at a gracing incidence angle of 30° with respect to the surface plane (red circles); see Supplementary Notes 2 and 4 for details.

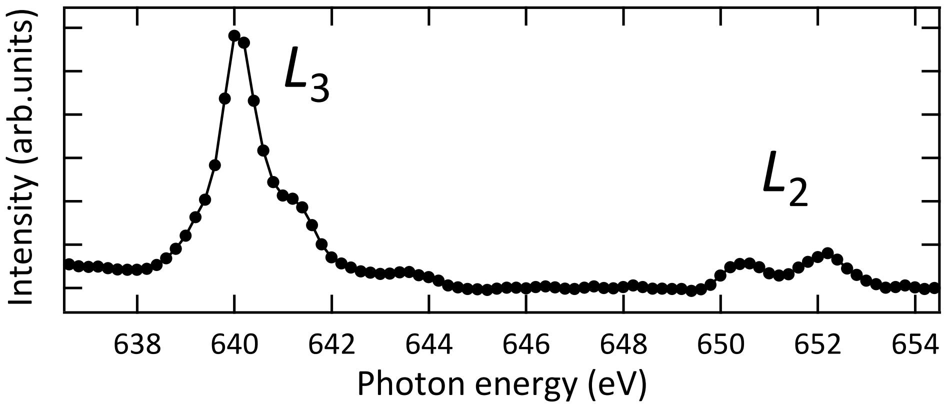

Supplementary Figure 7: **Absorption spectrum measured for the $(Bi_{1-x}Mn_x)_2Se_3$ sample doped with 8% Mn using X-PEEM.** The spectrum is obtained across the Mn $L_{2,3}$-edges from the same region of the sample shown in the XMCD images of Supplementary Fig. 8 below and plotted as the result of the sum of two absorption spectra recorded with opposite light helicity; see also Supplementary Note 3.

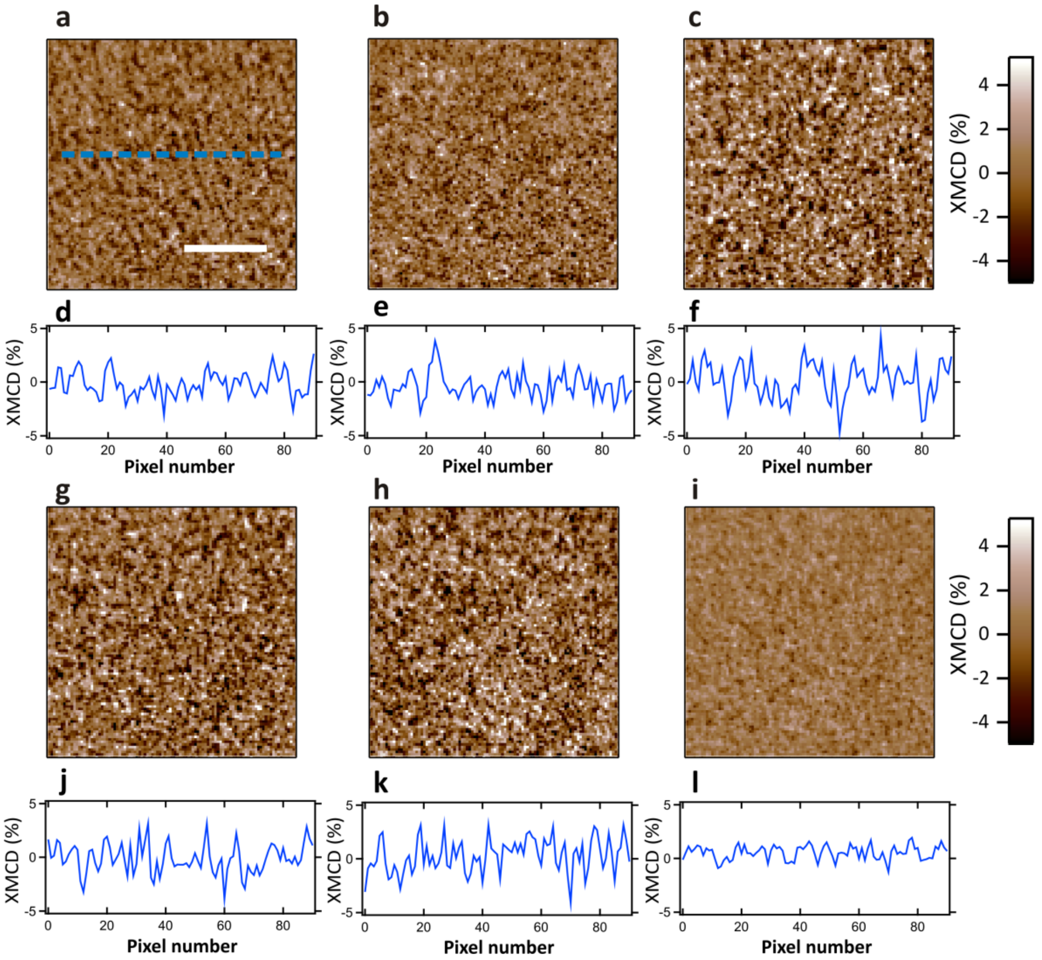

Supplementary Figure 8: **A sequence of XMCD-PEEM measurements.** (a-l) XMCD-PEEM measurements for the $(Bi_{1-x}Mn_x)_2Se_3$ sample doped with 8% Mn. (a, b, c, g, h) XMCD images obtained at the Mn L_3 -edge after pre-edge subtraction. Each XMCD consisted of 160 images (3 seconds integration time each). Panels (d, e, f, j, k) show the corresponding XMCD profiles across the same line [blue dotted line on panel (a)]. Panels (i) and (l) show the average XMCD and XMCD profile, respectively. The scale bar (horizontal white-solid line) shown in (a) is 500 μm and applies to all panels. Note the absence of correlation between the different individual measurements as well as the contrast decrease after averaging, indicating absence of ferromagnetic domains at room temperature; see Supplementary Note 3 for more details.

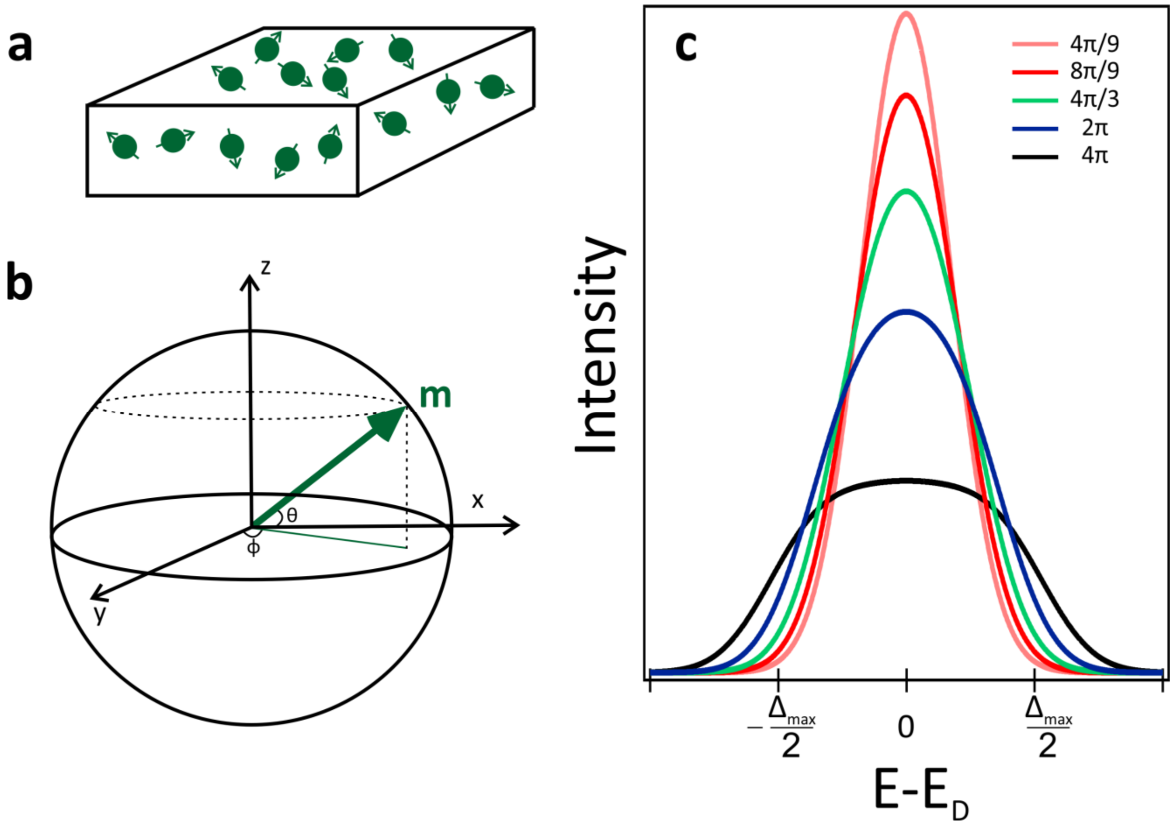

Supplementary Figure 9: **Simulations of spectral weight at the Dirac point.** (a) Schematic of a topological insulator hosting bulk magnetic impurities (green spheres) with random magnetic moments represented by green arrows. (b) A random magnetic moment in our simplified model is illustrated as a green arrow in the three-dimensional space. The in- and out-of-plane projection of the magnetic moment varies only with the polar angle θ and a randomized collection of magnetic impurities covers the whole polar and azimuthal (ϕ) range. The dashed line indicates the in-plane projection of the magnetic moment while the dashed circle encloses the full solid angle covered by a specific polar value. (c) Evaluation of the spectral weight using our model for different constraints in the magnetic moment orientation, plotted as a function of the energy away from the Dirac point ($E-E_D$). The constraints are chosen in accordance with the results of our magnetic characterization, which reveals either preferentially-oriented in-plane magnetic moments below T_c or random orientations of the Mn magnetic moments above T_c. The black curve corresponds to a fully randomized population after integration across all possible values of θ and ϕ. Blue, green, red and pink curves correspond to randomized magnetic moments constrained by symmetric polar angles with respect to the *x-y* plane which add up to the total solid angle indicated in the legend.

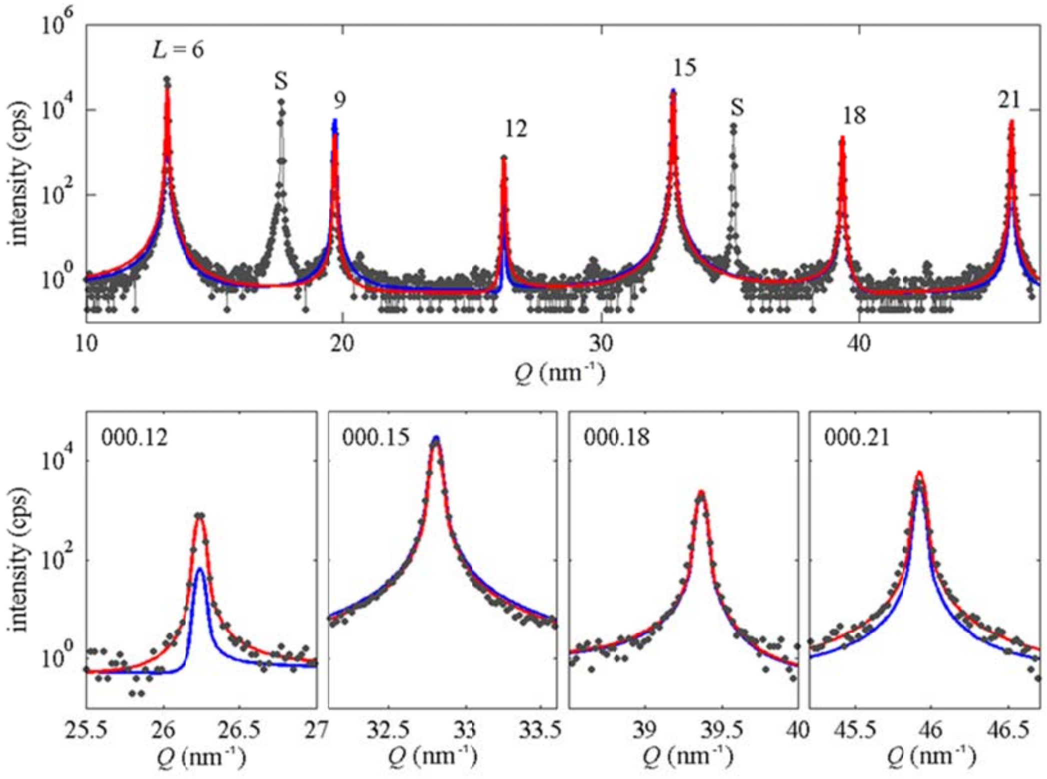

Supplementary Figure 10: **X-ray diffraction curves of the pure Bi_2Se_3 film.** The measured curves are shown as points and the simulations as lines. The upper panel shows the whole diffraction curve, the lower panels depict individual diffraction maxima. “S” in the upper panel denotes the substrate peaks, *L* are the orders of the diffraction peaks of Bi_2Se_3. The blue (red) curves depict the simulation results assuming nominal (changed) bilayer distances, respectively; see Supplementary Note 5 for details.

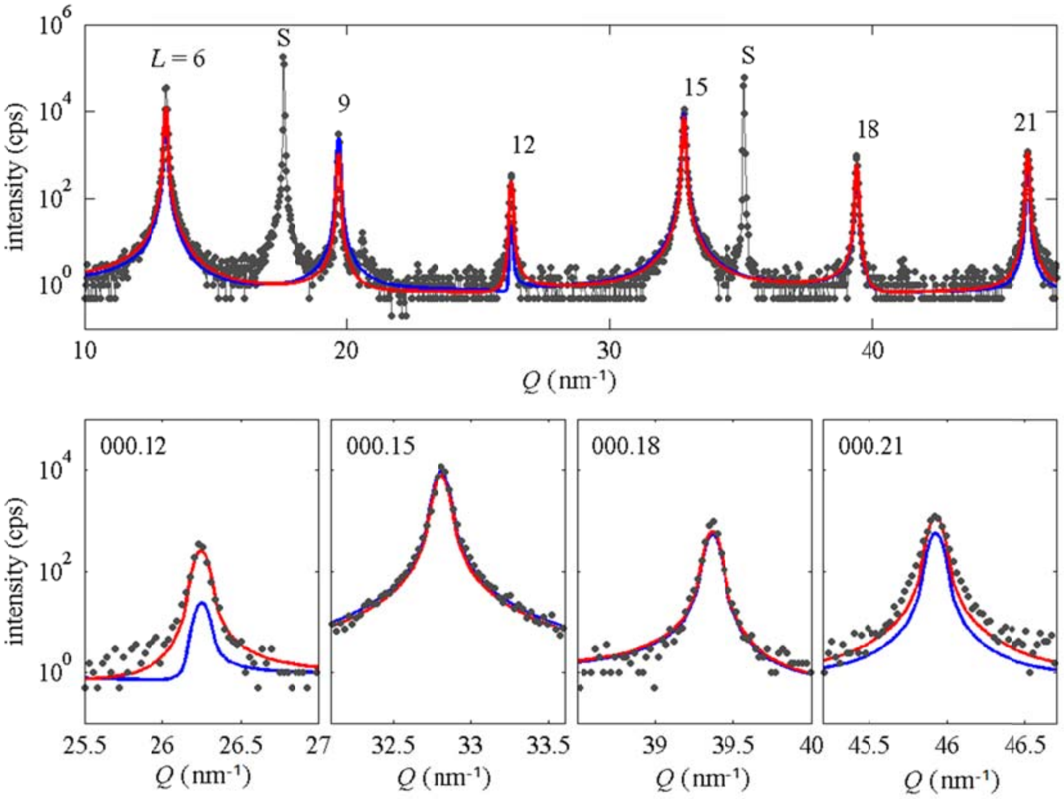

Supplementary Figure 11: **X-ray diffraction curves of the 2% Mn-doped Bi_2Se_3 film.** The measured curves are shown as points and the simulations as lines. The upper panel shows the whole diffraction curve, the lower panels depict individual diffraction maxima. “S” in the upper panel denotes the substrate peaks, *L* are the orders of the diffraction peaks of Bi_2Se_3. The blue (red) curves depict the simulation results assuming nominal (changed) bilayer distances, respectively; see Supplementary Note 5 for details.

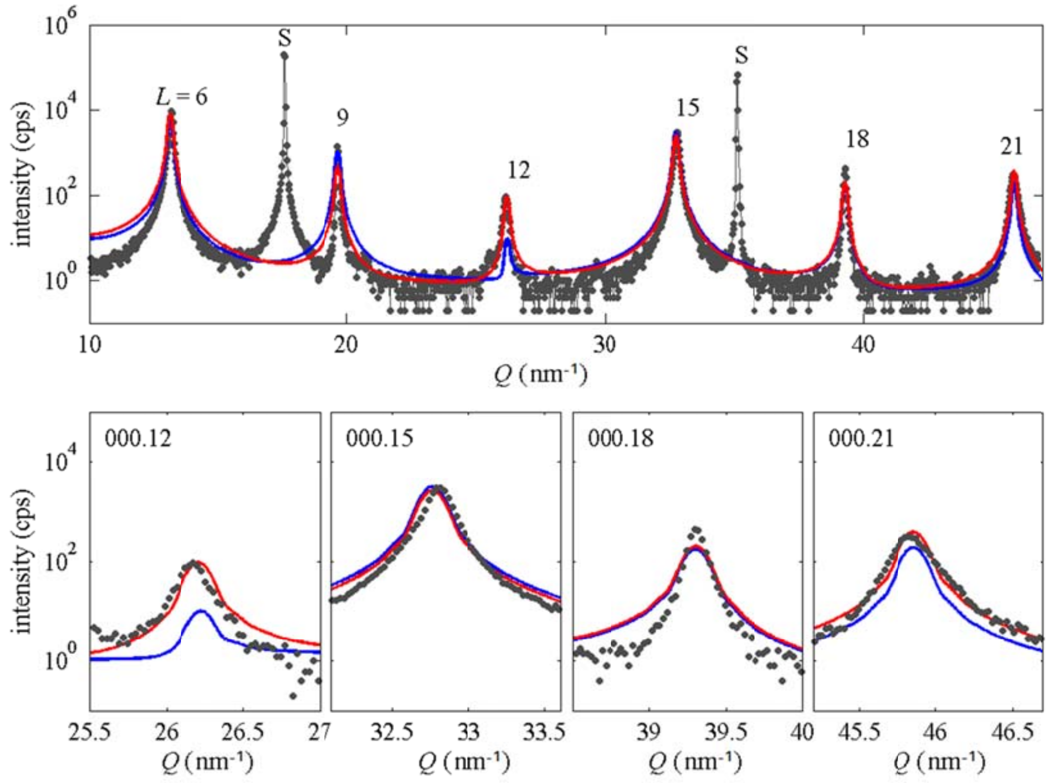

Supplementary Figure 12: **X-ray diffraction curves of the 4% Mn-doped Bi_2Se_3 film.** The measured curves are shown as points and the simulations as lines. The upper panel shows the whole diffraction curve, the lower panels depict individual diffraction maxima. “S” in the upper panel denotes the substrate peaks, *L* are the orders of the diffraction peaks of Bi_2Se_3. The blue (red) curves depict the simulation results assuming nominal (changed) bilayer distances, respectively; see Supplementary Note 5 for details.

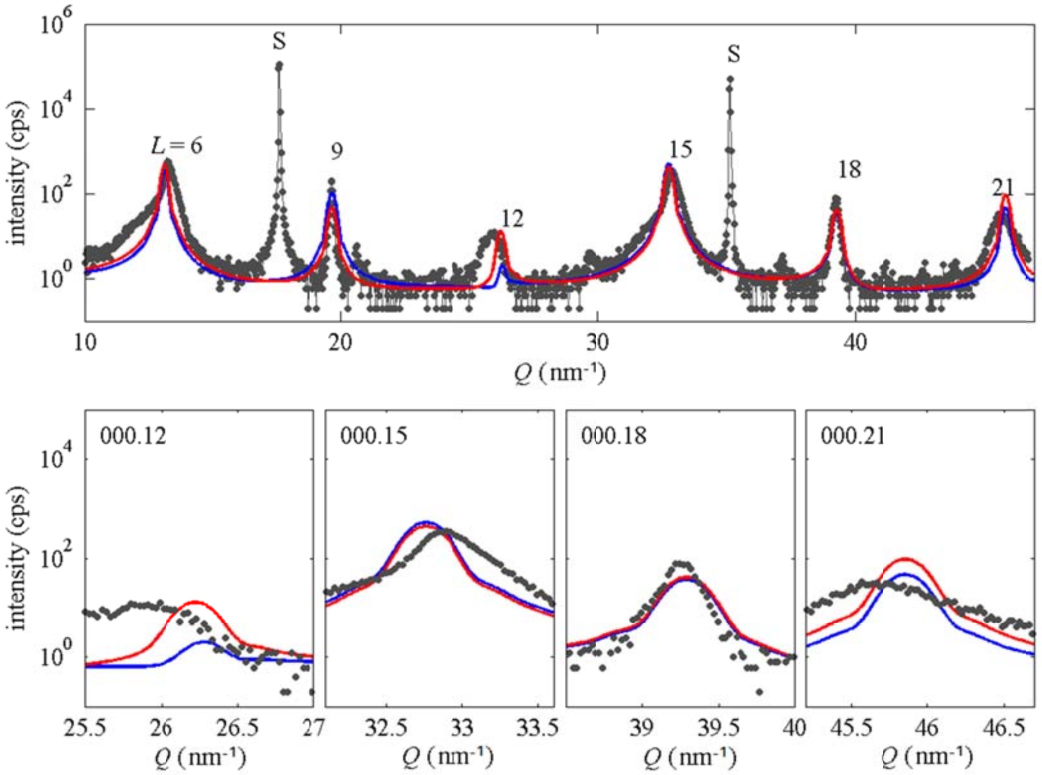

Supplementary Figure 13: **X-ray diffraction curves of the 8% Mn-doped Bi_2Se_3 film.** The measured curves are shown as points and the simulations as lines. The upper panel shows the whole diffraction curve, the lower panels depict individual diffraction maxima. "S" in the upper panel denotes the substrate peaks, *L* are the orders of the diffraction peaks of Bi_2Se_3. The blue (red) curves depict the simulation results assuming nominal (changed) bilayer distances, respectively; see Supplementary Note 5 for details.

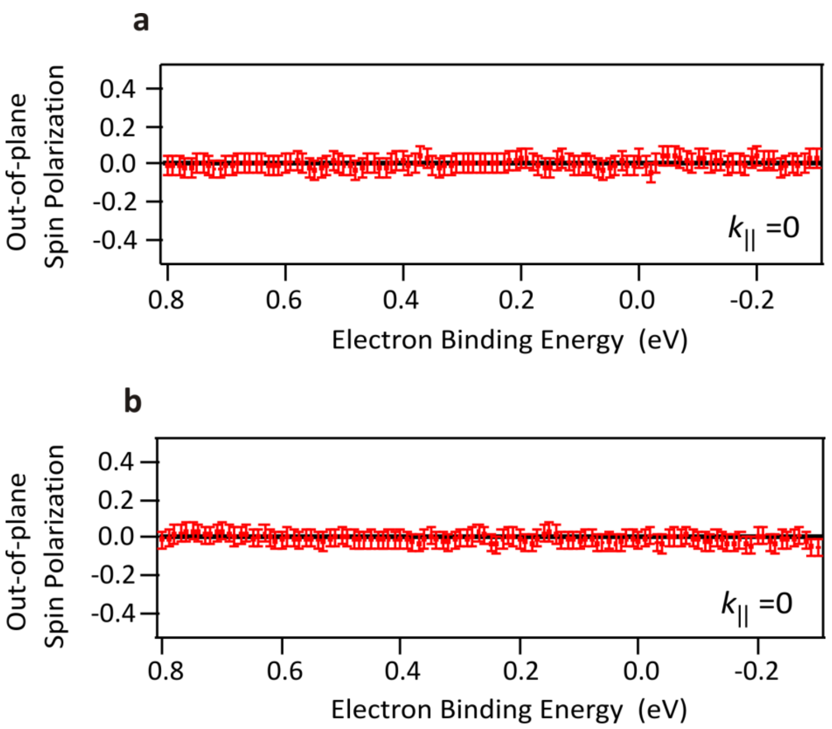

Supplementary Figure 14: **Spin-resolved measurements.** Out-of-plane spin polarization measurements obtained at zero momentum ($k_{||}$=0) from (a) the $(Bi_{1-x}Mn_x)_2Se_3$ sample with 8% Mn content (x = 0.08) and (b) the $(Bi_{1-x}In_x)_2Se_3$ sample with 2% In content (x = 0.02). No spin polarization perpendicular to the surface is observed in both cases. Measurements in (a) were taken at 20 K, while measurements in (b) at room temperature. In the case of the 8% Mn-doped Bi_2Se_3 sample, a magnetic field of +0.3 T was applied perpendicular to the surface plane at 20 K right before the acquisition of the data (similar results were obtained at room temperature).

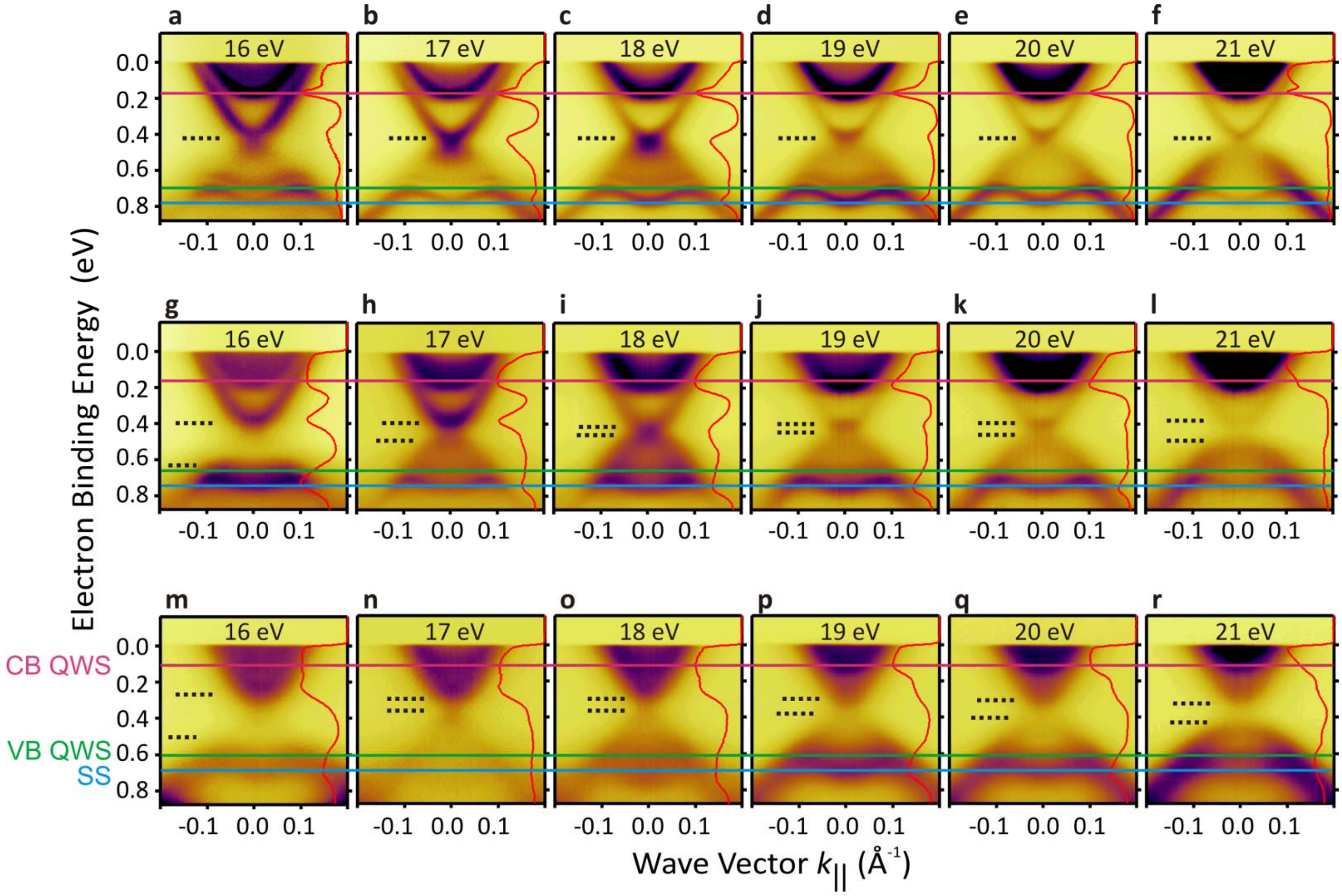

Supplementary Figure 15: **Photon-energy dependence of the surface gap.** Data as in Fig. 5 of the main text for more photon energies. Electronic band dispersions of (a-f) undoped Bi_2Se_3, (g-l) 2% and (m-r) 8% Mn-doped Bi_2Se_3. The photon energy changes between 16 and 21 eV as indicated in each panel. The red curves are the corresponding energy-distribution curves in normal-emission. The conduction band (CB) and valence band (VB) quantum-well states (QWS) are used to track changes of the bulk band gap. The M-shaped band at higher binding energy has been assigned to a surface state (SS) [5]. The SS may not track the band bending [5] but it tracks here clearly the Mn-induced hole doping. We rely, therefore, on the positions of the most intense CB QWS and VB QWS as observed for similar photon energies before [5]. Here, the separation between CB QWS and VB QWS stays constant within a few meV for 18 eV and within 20 meV when other photon energies are taken into account (horizontal blue, green and pink solid lines). This means that the bulk band gap does not change due to the Mn. The horizontal black-dashed lines in each panel highlight the minimum size of the surface band gap after taking into account the contribution from the linewidth broadening.

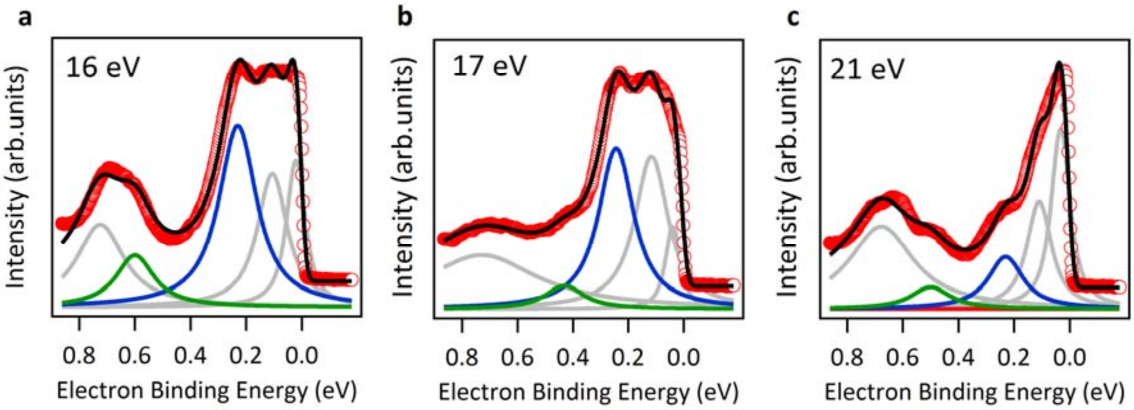

Supplementary Figure 16: **Surface band gap at different photon energies.** (a-c) Few-selected fits (solid black lines) of the energy-distribution curves (EDCs) at zero momentum ($k_{||}$=0, red circles) extracted from the ARPES dispersions shown in Supplementary Fig. 15 for the $(Bi_{1-x}Mn_x)_2Se_3$ sample doped with 8% Mn, at photon energies of (a) 16 eV, (b) 17 eV and (c) 21 eV and 12 K temperature. A variation of the surface band gap with photon energy is observed. Similar fits were done for other photon energies and Mn concentrations. The fitting procedure and the representation is the same as the one used in Supplementary Fig. 2. The fitted Lorentzian peaks shown in blue (green) color correspond to the minimum (maximum) of the upper (lower) Dirac cone. The minimum size of the surface band gap extracted at low photon energies is consistent with the one obtained at 50 eV. For details on the fitting procedure, see Supplementary Note 1.

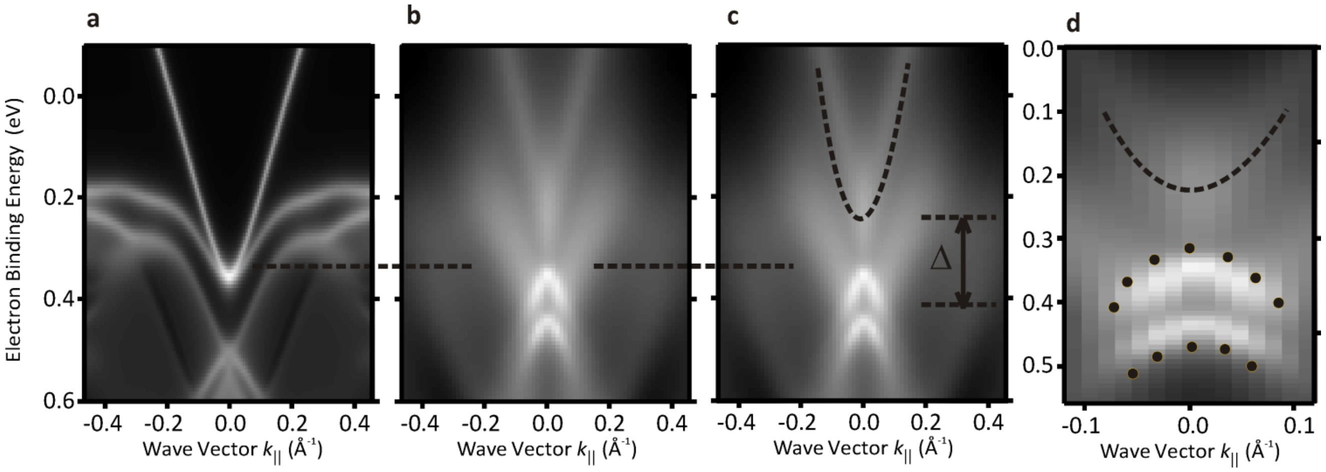

Supplementary Figure 17: **One step-model photoemission calculations.** (a-d) Results of the one step-model photoemission calculations for (a) Bi_2Se_3 and (b-d) 8% Mn-doped Bi_2Se_3 at 50 eV photon energy (see Supplementary Note 6 for details). In (a), the binding energy position of the Dirac point is denoted with a horizontal black dashed line which expands into the calculations shown in (b) and (c), where pronounced contributions from Mn-derived impurity resonances of strong *d*-character are clearly observed. Note that the calculations overestimate the intensity of the Mn-derived states as compared to the experiment. The reason is that transition matrix elements describing *d-p* or *d-f* transitions from *d*-like metals in our calculations are typically one order of magnitude larger than for *p-s* or *p-d* transitions from insulator or semiconductor materials. (c) Similar results as in (b), highlighting a gap opening of Δ~ 150 meV. Note that the gap is only well defined for the upper Dirac cone and with respect to the original Dirac point in Bi_2Se_3. (d) Zoom-in into the dispersion of the resonance states. The points (black circles) are extracted from the resonant photoemission experiment shown in Fig. 7f of the main text, revealing qualitative agreement between experiment and calculations.

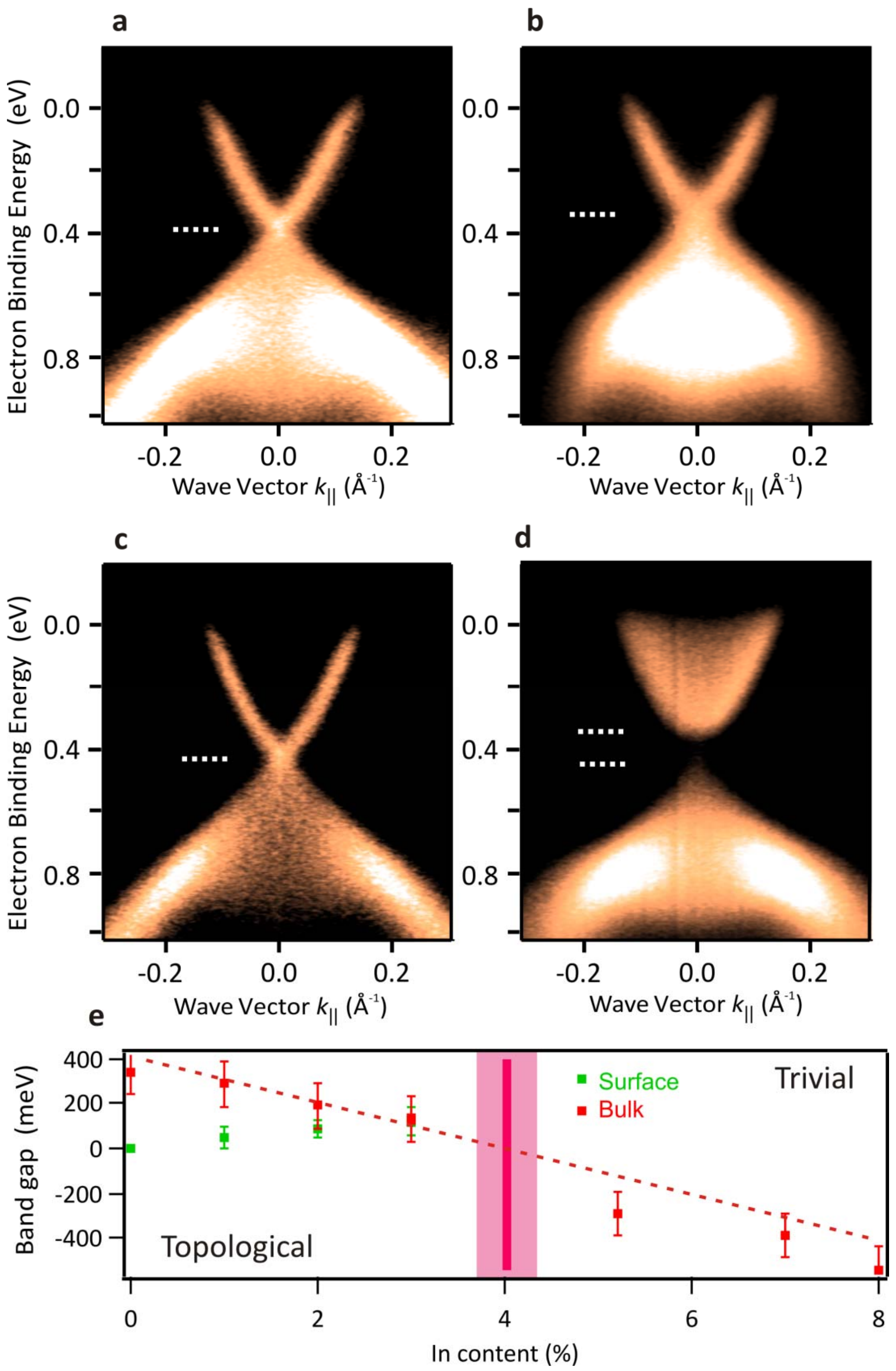

Supplementary Figure 18: **Control experiments using nonmagnetic Sn and In impurities.** (a, b) ARPES spectra obtained at room temperature from 0.4 μm thick films of (a) Bi_2Se_3 and (b) $(Bi_{1-x}Sn_x)_2Se_3$ doped with 4% Sn. (c,d) Corresponding ARPES spectra obtained from bulk single crystals of (c) undoped Bi_2Se_3 and (d) $(Bi_{1-x}In_x)_2Se_3$ doped with 2% In. The horizontal white-dashed lines in (a-c) mark the Dirac point position, while in (d) emphasize the surface band gap. (e) Dependence of the bulk band gap (red solid symbols) and surface band gap (green solid symbols) for the In-doped Bi_2Se_3 samples across different points of the topological to trivial quantum-phase transition (see Supplementary Note 7 for details). The region of the inversion point is marked by a red colored area and a vertical red-solid line. The red-dashed line is a guide to the eye.

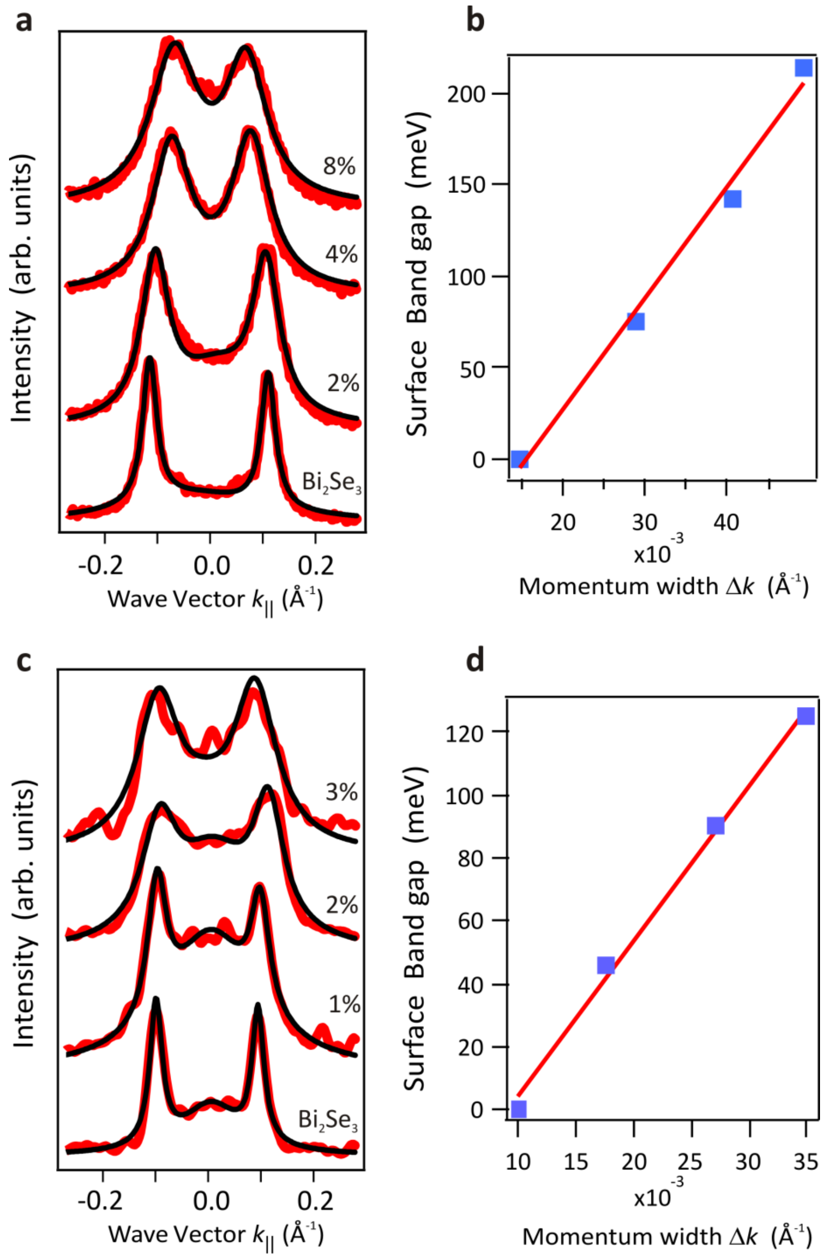

Supplementary Figure 19: **Momentum broadening as a function of doping.** (a-d) Analysis of the momentum broadening in the immediate vicinity of the Fermi level for (a, b) Mn-doped Bi_2Se_3 films and (c, d) In-doped Bi_2Se_3 bulk crystals. In (a) and (c), we show the momentum-distribution curves extracted from the ARPES spectra (red solid lines) and the corresponding fits (black solid lines). Panels (b) and (d) display the dependence of the surface band gap with the momentum broadening for both Mn and In-doped samples, respectively (see Supplementary Note 7 for details). The red lines are the result of a linear fit to the data (blue-colored squares).

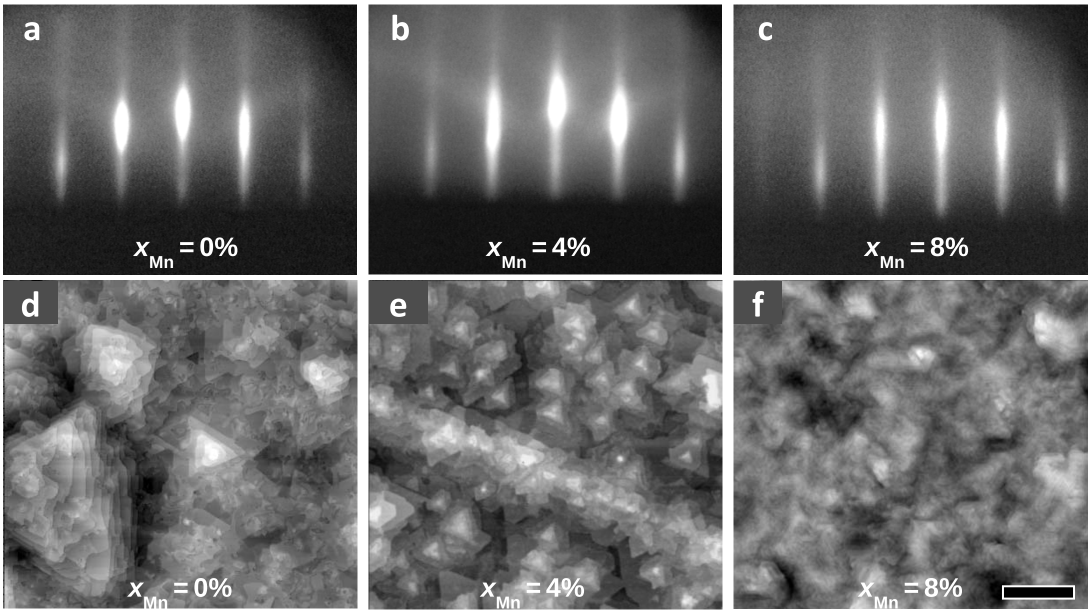

Supplementary Figure 20: **Sample characterization.** (a-c) Reflection high-energy electron diffraction patterns and (d-f) atomic force microscopy images of ~0.4 μm thick $(Bi_{1-x}Mn_x)_2Se_3$ epitaxial layers grown by MBE on BaF_2 (111) substrates for Mn concentrations of (a, d) 0 %, (b, e) 4 % and (c, f) 8% (See Supplementary Note 8 for details). The scale bar (horizontal black-line) shown in (f) is 1 μm and applies also to panels (d) and (e).

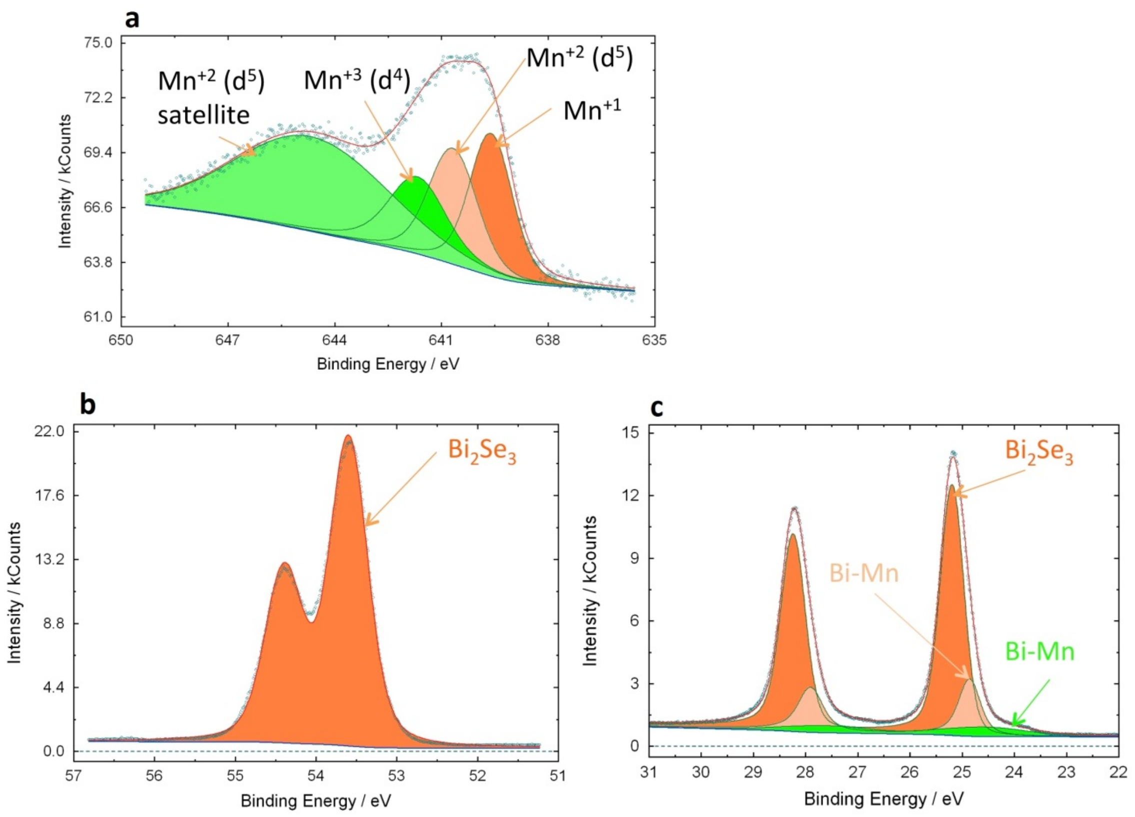

Supplementary Figure 21: **Core-level photoemission measurements.** (a-c) Core-level photoemission spectra from the $(Bi_{1-x}Mn_x)_2Se_3$ sample with the Mn content of 8% (x=0.08). The spectra are acquired at electron kinetic energy of 70 eV and correspond to (a) Mn 2p, (b) Se 3d and (c) Bi 5d core levels; see Supplementary Note 9 for more details.

x_{Mn} (%)	c (nm)
0	2.8736 ± 0.0003
2	2.8742 ± 0.0003
4	2.8779 ± 0.0005
8	2.8865 ± 0.0010

Supplementary Table 1: **Estimated c-lattice parameters for different Mn concentrations.** The table shows the values of the c-lattice parameters as determined from the positions of the diffraction maxima in Supplementary Figs. 10-13 for different Mn concentrations (see Supplementary Note 5 for details). The errors are obtained from the standard deviations in the fitted peak positions.

x_{Mn} (%)	$\Delta d_{Se\text{-}Se}$ (nm)	$\Delta d_{Se\text{-}Bi}$ (nm)
0	0.010 ± 0.005	-0.010 ± 0.005
2	0.020 ± 0.005	-0.015 ± 0.010
4	0.030 ± 0.005	-0.020 ± 0.010
8	0.030 ± 0.010	-0.020 ± 0.015

Supplementary Table 2: **Estimated bilayer distances for different Mn concentrations.** We tried to improve the correspondence of the measured and simulated diffraction curves by changing the values of the bilayer distances keeping their sum $d_{Se\text{-}Se}+2(d_{Se\text{-}Bi}+d_{Bi\text{-}Se})$ within one quintuplet constant and equal to $c/3$, since three quintuplets create one Bi_2Se_3 elementary unit cell. The best correspondence was achieved by changing the Se-Se distance by $\Delta d_{Se\text{-}Se}$ and that of the first neighboring Se-Bi bilayer by $\Delta d_{Se\text{-}Bi}$. The errors are estimated from the standard deviations between simulations and experiments; see Supplementary Note 5 for details.

Supplementary Note 1: Determination of the surface band gap

To determine the size of the surface band gap for different Mn concentrations, we have fitted the experimental energy-distributions curves (EDCs) at zero momentum extracted from the ARPES spectra acquired at different photon energies. In Supplementary Fig. 2, we show the fit results (black solid lines) of the EDCs (red circles) acquired at 50 eV photon energy and 12 K temperature as an example. Panels (a) to (d) display the fit results from 2 up to 8% Mn, respectively. The size of the surface band gap obtained from the fits is shown in Supplementary Fig. 2d. The error bars correspond to the uncertainty of determining the energy position of the band dispersions, and are estimated from the standard deviations of the peak positions over several fitting cycles. The size of the surface band gap in Supplementary Figs. 2a-c is determined from the energy separation between the fitted Lorentzian peaks shown in blue (green) color, which are located at the energy minimum (maximum) of the upper (lower) Dirac cone. We point out that a lower limit of the surface band gap can be obtained from the ARPES spectra after considering the contribution from the linewidth broadening. The lower limit of the surface band gap is approximately represented by horizontal dashed lines around the region of the gap in the ARPES figures shown in the present work, while in Supplementary Fig. 2d we plot the actual gap size as these are the absolute values to which we refer in the main text. In the fits of Supplementary Figs. 2a-c, other Lorentzian peaks shown in gray color are contributions from the bulk-valence bands. To extract the energy positions, the experimental ARPES spectra were fitted by a sum of Lorentzian functions plus a background. A typical spectrum containing N number of peaks is fitted by a function involving a convolution of the form:

$$I(E,k) = \left[f(E,T) \cdot \sum_{i=1}^{N} M_i^2 \cdot A(E_i, w_i) + B(E) \right] \otimes G(E)$$

Where E_i, ω_i, and the matrix elements M_i are fitting parameters corresponding to the binding energy, width, and intensity of each Lorentzian peak, and f(E,T) is the Fermi function. The spectral function $A_i(E_i,\omega_i)$ is approximated by Lorentzian functions, and B(E) is assumed to be a Shirley-like background [1]. The full width at half maximum (FWHM) of the Gaussian slit function G(hv) corresponds to the total energy resolution ΔE of the experiment, which is photon-energy dependent.

Supplementary Note 2: Field-cooling, zero-field cooling and XMCD above T_c

In Supplementary Fig. 5, we show field-cooling (FC) and zero-field-cooling (ZFC) measurements obtained using SQUID magnetometry for the Mn-doped Bi_2Se_3 samples with (a) 4% and (b) 8% Mn content. The temperature dependence of both the in-plane and out-of-plane components of the magnetization is shown. The FC measurements are taken under an applied magnetic field of 10 mT parallel and perpendicular to the surface. It is clearly seen that the FC and ZFC measurements follow each other for each Mn concentration. In addition, the measured magnetization is always larger or equal for an in-plane magnetic field as compared to an out-of-plane magnetic field at all temperatures. Therefore, there is no sudden change in the orientation of the easy axis above T_c, while below T_c the easy axis is clearly parallel to the sample surface.

We point out that the SQUID results are sensitive to the magnetization of the bulk, and thus cannot generally be used to rule out static short-range magnetic order above T_c at the surface. Nevertheless, we emphasize that one of the main outcomes of our magnetic characterization is that both surface and bulk have basically the same magnetic properties (note the small surface T_c enhancement by ≤ 4 K).

In order to further address the possibility of static short-range magnetic order above T_c at the surface, in Supplementary Fig. 6 we show XMCD measurements taken for 8% Mn-doped Bi_2Se_3 at a temperature of 20 K and under an applied magnetic field of -2T parallel (red circles) and perpendicular to the sample surface (black solid lines). Similar results were obtained for 4% Mn doping. Note that even for an applied magnetic field of 2 T, below T_c the system is not magnetically saturated irrespective of the Mn concentration (see, e.g., inset of Fig. 4b of the main text), thus it cannot be saturated at 20 K. Moreover, the x-ray beam impinged the sample in normal (black solid lines) and grazing incidence (red circles), meaning that the XMCD measurements shown in Supplementary Fig. 6 are sensitive to the out-of-plane and in-plane components of the magnetization, respectively. In the presence of short-range static magnetic order, the application of a magnetic field is crucial to verify if above T_c a preferential orientation of the Mn magnetic moments perpendicular to the surface exists, which is one of the preconditions for a local band gap opening. However, above T_c such a preferential orientation is neither observed in our XMCD nor in our SQUID results described above, indicating that short-range static magnetic order at the surface or in the bulk with preferentially oriented Mn magnetic moments perpendicular to the surface is not responsible for the opening of the large surface band gaps that we observe. Taking into account that under an applied magnetic field above T_c there is no such preferential orientation, and that the corresponding XMCD signal without an applied magnetic field is zero, we can conclude that above T_c the system is characterized by random orientations of the Mn magnetic moments. We have to stress that in ARPES, static magnetic moments pointing along random directions within the probed area can be viewed as a superposition of different photoemission signals that are spatially averaged (gapped vs. non-gapped states). As canted magnetic moments introduce a gap which varies in size depending on the magnitude of their perpendicular component [6], it is straightforward to demonstrate that in the case of random Mn magnetic moments most of the contributions to the ARPES intensity will be concentrated near the Dirac point (see Supplementary Note 4 for further discussion and analysis).

Supplementary Note 3: Magnetic imaging at the Mn L_3-edge

To additionally address whether magnetic domains might form, we performed x-ray photoelectron emission microscopy (X-PEEM) experiments at room temperature. The circularly-polarized (90% polarization) x-ray beam impinged the sample at a gracing incidence angle of 16°. With such geometry the experiment is sensitive to both, in-plane and out-of-plane components of the magnetization since both present a projection along the x-ray beam propagation direction. The lateral resolution is ~20 nm. For magnetic imaging, we exploited the element and magnetic sensitivity of x-ray circular magnetic dichroism (XMCD). In Supplementary Fig. 7, we show a typical absorption spectrum measured for the $(Bi_{1-x}Mn_x)_2Se_3$ sample doped with 8% Mn using X-PEEM. The spectrum is obtained across the Mn $L_{2,3}$-edges from the same region of the sample shown in the XMCD images of Supplementary Fig. 8, and plotted as the result of the sum of two absorption spectra recorded with opposite

light helicity. The lineshape is consistent with the absorption spectra obtained without lateral resolution shown in the main text, revealing a predominant d^5 configuration.

In panels (a), (b), (c), (g) and (h) of Supplementary Fig. 8, we show a total of five consecutive XMCD-PEEM measurements, where the magnetic contrast from exactly the same region of the sample is shown. Each measurement consisted of 160 images (3 seconds integration time each); 80 at the Mn L_3-edge (40 per helicity) and 80 at the pre-edge (40 per helicity). After normalization to a bright-field the sequence was drift-corrected, and frames recorded using the same photon energy and helicity of the circularly-polarized light were averaged. In order to optimize the magnetic sensitivity, the pre-edge data was subtracted from that measured at the Mn L_3-edge. The Mn magnetic contrast is then plotted as the difference between the two resulting average images with opposite helicity (after pre-edge subtraction), divided by their sum. The results reveal that there is no correlation between the different images. This can also be clearly seen in the curves plotted below each XMCD image, which correspond to XMCD profiles extracted across the same line [blue dotted line drawn on panel (a)]. Panels (i) and (l) of Supplementary Fig. 8 show the XMCD-PEEM image and the corresponding XMCD profile that result from the average of all individual measurements, respectively. The XMCD contrast for the average XMCD image has decreased by more than a factor five, and lies below the ±1% range. The absence of correlation between measurements, as well as the contrast decrease after averaging indicates that the XMCD signal spans over statistical noise, i.e. there are no ferromagnetic domains with full or partial in-plane or out-of-plane magnetization.

Supplementary Note 4: Short-range magnetic fluctuations and Dirac point spectral weight

As discussed in Supplementary Note 2, our SQUID and XMCD measurements establish that above T_c short-range static magnetic order does not play a role in the bulk and at the surface, respectively. We have further supported this conclusion by XMCD-PEEM measurements revealing the absence of short-range inhomogeneous magnetic order, at least within the lateral resolution of our experiment. In this respect, we believe that the agreement and consistency of all individual experiments taken as a whole strongly supports our conclusions, rather than focusing on the individual outcome of a single experiment utilizing a specific technique.

Having said this, the only remaining possibility for a local band gap opening due to magnetism would be magnetic short-range fluctuations. However, we have to stress that for a local band gap opening to fully develop due to short-range magnetic fluctuations above T_c, one would again require a preferential orientation of the fluctuating magnetic moments perpendicular to the surface. In this scenario, the argumentation is very similar to the one given in Supplementary Note 2. By applying a magnetic field parallel and perpendicular to the surface above T_c, it is in principle possible to determine the existence of such an anisotropy using a slow probe such as XMCD. Our XMCD measurements shown in Supplementary Fig. 6, however, indicate that if short-range magnetic fluctuations are at play above T_c, these are not preferentially oriented perpendicular to the surface plane. We emphasize again that under the conditions of the measurements shown in Supplementary Fig. 6, the system is not magnetically saturated. Taking into account that above T_c and without an applied magnetic field the resulting XMCD signal disappears, if short-range magnetic fluctuations are present without an applied magnetic field, these are likely to be random. Moreover, based on the SQUID measurements shown in Supplementary Fig. 5, a

similar conclusion can be drawn in the case of magnetic fluctuations in the bulk. We point out that in ARPES, the effect of magnetic fluctuations would similarly result in a complex redistribution of the Dirac point spectral weight, regardless of whether ARPES is a slower or faster probe compared to the time scale of the magnetic fluctuations. The reason is that a large amount of fluctuating magnetic moments pointing along different directions within the probed area can be viewed as a superposition of different photoemission signals that are averaged in space and time (gapped vs. non-gapped states). These signals would emerge with different relative statistical weights depending on how long and which part of the fluctuating magnetic moments is preferentially oriented perpendicular or parallel to the surface. Without a preferential orientation of the fluctuating magnetic moments perpendicular to the surface, most of the contributions to the ARPES intensity will be concentrated near the Dirac point. Note that this is also true for static random Mn magnetic moments, where only the spatial average of the individual signals is important. Using a simplified model, it is possible to show that the same conclusion holds in the case of short-range random magnetic fluctuations.

For this purpose, we have developed an ergodic model to simulate the spectral weight in the vicinity of the Dirac point for a randomized population of Mn magnetic moments (Supplementary Fig. 9a). In our model, canted magnetic moments introduce a gap which varies in size from zero to Δ_{max} depending on the magnitude of their out-of-plane component [6] (Supplementary Fig. 9b). For simplicity, we have not considered the additional smearing of the Dirac point intensity along the momentum direction due to in-plane magnetic moments. We represent the electronic bands by Gaussian peaks with a full-width at half-maximum which is about 30% of the maximum value of the band-gap size located symmetrically with respect to the Dirac point when the gap opens. Note that our model works for any possible generic value of Δ_{max}. In addition, by increasing the broadening of the bands in our model, even when the surface-state linewidth is larger than the maximum possible gap, we obtain very similar results. Supplementary Fig. 9c depicts the evolution of the spectral weight at zero momentum ($k_{\parallel}$=0) from a fully randomized population of magnetic moments (black line) to configurations where the magnetic moments are restricted to polar angles θ around the *x-y* plane (colored lines), in correspondence with the results of our magnetic characterization. For the sake of clarity, a Shirley-like background has not been considered in this representation. The resulting spectral weight is obtained after integrating all allowed polar angles θ over the full range of azimuthal angles in accordance with the rotational symmetry of our problem (see Supplementary Fig. 9b).

We clearly observe that the magnetic moment randomization smears the intensity along the energy axis across the whole gap range. This is due to the fact that the intensity around the Dirac point spans over all possible gaps due to the weighted contributions from random magnetic moments. In particular, the randomization leads to the largest intensity around the Dirac point, while the weakest intensity appears away from the Dirac point near the maximum possible gap corresponding to a purely perpendicular moment, in contrast to our observations. Finally, we would like to stress that based on the results of Ref. 7, and taking into account the small predicted gap size from ab-initio calculations ($\Delta_{max} \sim$ 9 meV for Co on Bi_2Se_3 (Ref. 8) and $\Delta_{max} \sim$ 16 meV for Mn in Bi_2Te_3 (Ref. 9), it is difficult or almost impossible to anticipate static short-range magnetic order or short-range magnetic fluctuations as the reason for the large 100 meV gap observed in our work and in Ref. 10.

Supplementary Note 5: X-ray diffraction

From the x-ray diffraction data taken in symmetric $\omega/2\Theta$ scans we determined the *c*-lattice parameter and estimated also the changes in the bilayer distances within the Bi_2Se_3 quintuplets. In Supplementary Figs. 10 – 13 we present the measured and simulated $\omega/2\Theta$ scans (upper panels) as well as their details around the diffraction maxima 000.12 to 000.21 (lower panels). The values of the *c*-lattice parameters determined from the positions of the diffraction maxima are presented in Supplementary Table 1.

As expected, the c-lattice parameter increases with increasing Mn content. We have simulated the diffraction curves using standard x-ray kinematical diffraction theory, including the effects of partial polarization of the primary beam, absorption correction and the correction to the irradiated sample surface. The aim of the simulation was to compare the heights of the measured diffraction maxima with the theoretical prediction, since the heights are proportional to the square of the $(Bi_{1-x}Mn_x)_2Se_3$ structure factor that sensitively depends on the distance of individual bilayers.

In the simulations we assumed nominal bilayer distances relative to the measured *c* values, these relative distances were taken from the PDF-4 database [11], the results of the simulations are plotted by blue lines. As we demonstrated in our previous paper [12], the quasi-forbidden diffraction maximum 000.12 is extremely sensitive to the bilayer spacings. From our simulations it follows that even for pure epitaxial Bi_2Se_3, the individual bilayer distances in the unit cell do not correspond to the values in Ref. 11, which were determined from the diffraction data on bulk single crystals. We tried to improve the correspondence of the measured and simulated diffraction curves by changing the values of the bilayer distances keeping their sum $d_{Se\text{-}Se}+2(d_{Se\text{-}Bi}+d_{Bi\text{-}Se})$ within one quintuplet constant and equal to *c*/3, since three quintuplets create one Bi_2Se_3 elementary unit cell.

The best correspondence (yet not good enough) was achieved by changing the Se-Se distance by $\Delta d_{Se\text{-}Se}$ and that of the first neighboring Se-Bi bilayer by $\Delta d_{Se\text{-}Bi}$; their values are summarized in Supplementary Table 2.

The modified calculation results are denoted by red curves in Supplementary Figs. 10 – 13. With increasing Mn content the diffraction maxima get broader and for the highest Mn content of 8% the maxima are deformed so that an exact determination of the Δd values is not possible. However, the table clearly indicates a possible expansion of the van der Waals Se-Se bilayer due to the Mn atoms. The broadening of the maxima is caused with a deterioration of the crystal quality with increasing x_{Mn}. The nature of the structure defects is not clear, most likely planar defects like stacking faults or Bi-Bi bilayers randomly placed in the lattice are responsible for this broadening. In the simulations this effect was included by decreasing the mean number of quintuple layers creating one coherently diffracting domain (see Ref. 12 for details).

Supplementary Note 6: One-step model calculations

Using the results of ab-initio theory within the coherent potential approximation as an input, we have performed additional calculations within the one-step model of photoemission [13]. The calculations are based on multiple scattering theory including wave-vector and energy-dependent transition matrix elements [14]. We use a fully relativistic version which is part of the Munich SPR-KKR program package [15], with spin-orbit coupling included by solving the

four-component Dirac equation. In particular, the one-step model photoemission calculation for Mn-doped Bi_2Se_3 was done using the implemented version for disordered magnetic materials [16], while the calculation for the undoped Bi_2Se_3 system was done using the implemented version for ordered materials in its spin-density matrix formulation [17].

In Figure 17, we show the results of the one step-model photoemission calculations for (a) undoped Bi_2Se_3 and (b-d) 8% Mn-doped Bi_2Se_3 at 50 eV photon energy. In (a), the binding energy position of the Dirac point is denoted with a horizontal black dashed line which expands into the calculations shown in (b) and (c), where pronounced contributions from Mn-derived impurity resonances of strong *d*-character arising from hybridized t_{2g} and e_g orbitals are clearly observed. The calculations clearly overestimate the intensity of the Mn-derived states as compared to the experiment. The reason is that transition matrix elements describing *d-p* or *d-f* transitions from d-like metals in our calculations are typically one order of magnitude larger than for *p-s* or *p-d* transitions from insulator or semiconductor materials. Note that such a disagreement regarding the intensities of Mn-derived states in the calculations does not alter our conclusions or the physics underlying the origin of the surface band gap. In panel (c) of Supplementary Fig. 17, we show similar results as in panel (b), highlighting a gap opening of $\sim$ 150 meV. Note that the gap is only well defined for the upper Dirac cone and with respect to the original Dirac point in Bi_2Se_3. In Supplementary Fig. 17d, we show a zoom-in into the dispersion of the resonance states. The points (black circles) are extracted from the resonant photoemission experiment shown in Fig. 7f of the main text, revealing qualitative agreement between experiment and calculations.

We attribute the additional deviations between experiment and theory to the fact that we use an atomic-sphere approximation in the electronic structure calculation which, moreover, leads to an overestimation of the surface contribution. Considering that the calculated dispersions are very sensitive to the geometrical structure of the first few layers, a full-potential electronic structure calculation would improve the agreement. Nonetheless, we emphasize again that these deviations do not prevent us from capturing the main physics underlying the origin of the gap in the calculations. In the framework of a fully self-consistent full-potential electronic structure calculation an even closer agreement between experiment and theory concerning the electronic structure is expected. To obtain an even higher level of agreement in a spectroscopical calculation, a fully relativistic full-potential photoemission theory that is intimately based on the corresponding input from a self-consistent SPR-KKR half-space calculation would be needed, which is still an important task for future studies. It would be a very important step forward to implement such a photoemission theory in the framework of the one-step model, as it has not been done so far. In this way, future spectroscopical calculations could probably deal with full-potential based electronic structure inputs.

Supplementary Note 7: Non-magnetic impurities

As mentioned in the main text, we have performed additional control experiments using nonmagnetic Sn and In impurities in Bi_2Se_3. These experiments have been performed for both epitaxial films and bulk single crystals, from which we have obtained similar results for both types of impurities. In Supplementary Fig. 18 we show ARPES spectra obtained at room temperature from ~0.4 μm thick films of (a) Bi_2Se_3 and (b) $(Bi_{1-x}Sn_x)_2Se_3$ doped with 4% Sn. Both films are grown on BaF_2 (111) substrates, similar to the case of the Mn-doped films. We have obtained similar results in a wide range of Sn concentrations of up to 8%. In panels (c)

and (d), we show the corresponding ARPES spectra obtained from thick bulk single crystals of (c) undoped Bi_2Se_3 and (d) $(Bi_{1-x}In_x)_2Se_3$ doped with 2% In. We have also examined a wide range of In concentrations of up to 8%.

It can be clearly seen that after doping with Sn (Supplementary Fig. 18b), no surface band gap opens at the Dirac point, a result that we can confirm up to high-levels of Sn concentrations. On the other hand, our Bi_2Se_3 sample doped with 2% In (Supplementary Fig. 18d) clearly exhibits the opening of a large surface band gap, which is of the order of 100 meV, a result that is completely in contrast to the case of the sample doped with 4% Sn concentration. Interestingly, the size of the surface band gap for the In-doped Bi_2Se_3 sample is of the same order of the gap observed for the 8% Mn doped Bi_2Se_3 sample. This indicates that the concentration range at which the large surface gap develops varies from dopant to dopant, a fact which based on the ideas proposed in papers like Refs. 18,19 might be associated with the impurity-dependent strength of the Coulomb U, regardless whether the dopant is magnetic or not.

As mentioned in the main text, we emphasize that in the case of In-doped Bi_2Se_3 samples a topological to trivial quantum-phase transition is known to exist (see, e.g., Ref. 20). Therefore, we have examined the bulk band gap in a wide range of In concentrations across the quantum-phase transition. The results are shown in Supplementary Fig. 18e, where in addition to the bulk band gap (red solid symbols) we plot the size of the surface band gap (green solid symbols). The size of the bulk band gap is obtained from photon-energy dependent ARPES measurements as they track the dispersion of the bulk-bands perpendicular to the surface. In the ARPES measurements, we typically observe that the bulk band gap decreases progressively up to concentrations of about 5% In. The ARPES spectra reveal that above 5% In the bulk band gap increases again, and finally reaches a value above 0.4 eV for 8% In doping which is larger than the bulk band gap in pure Bi_2Se_3. The bulk band gap extracted from the ARPES spectra reveals a clear minimum somewhere around 4 to 5% In concentration. This can be obtained with some degree of accuracy by a parabolic fit of the bulk band dependence with In concentration. Moreover, the concentration at which the bulk band inversion occurs fits well with our preliminary optical measurements. Thus, in Supplementary Fig. 18e, we reverse the sign of the bulk band gap for In concentrations above 5% to illustrate the inversion of the bulk bands more clearly, and mark the region of the inversion point by a red colored area. The changes are also highlighted by a red-dashed line, which is a guide to the eye, as the bulk band dependence with In concentration might not be necessarily linear. These results are very reproducible, cleavage-to-cleavage, and confirm the existence of an inversion point, in addition to the fact that the large surface gap opening observed in Supplementary Fig. 18d for 2% In doped-Bi_2Se_3 samples occurs in the topological side of the quantum-phase transition. Our control experiments demonstrate the existence of a novel mechanism for surface band gap opening which is not directly connected to long-range or local magnetic properties.

Another interesting aspect is related to the linewidth broadening of the surface states near the Fermi level. If we compare the ARPES spectra shown in panels (a) and (c) of Supplementary Fig. 18 for Bi_2Se_3 epitaxial layers and bulk crystals, respectively, we can observe that the linewidth of the MBE-grown films is larger than the one of the bulk crystals, which is always the case for samples measured under the same experimental conditions. We attribute the additional broadening in the MBE-grown films to surface disorder, since disorder degrades the surface quality. At the same time, this means that surface disorder

does not open a surface band gap at the Dirac point, otherwise it should have been observed in Supplementary Fig. 18a for pure Bi_2Se_3 films. Moreover, note that at 4% Sn doping in Supplementary Fig. 18b the broadening does not increase substantially, in contrast to the case of 2% In doping (Supplementary Fig. 18d), where in addition to the opening of a surface band gap the broadening increases drastically. These differences suggest that an important contribution to the linewidth broadening originates from the surface band gap opening. This result motivates us to examine more closely the relation between the linewidth broadening and the size of the surface gap opening in both In-doped and Mn-doped samples, as the additional broadening can also be observed for the Mn-doped Bi_2Se_3 films.

In Supplementary Fig. 19, we show the results of the linewidth analysis, which we extract as momentum broadening Δk for (a, b) Mn-doped Bi_2Se_3 films and (c, d) In-doped Bi_2Se_3 bulk crystals. We have fitted the momentum-distribution curves (MDCs) in the immediate vicinity of the Fermi level for different compositions. In Supplementary Figs. 19a,c we show the experimental MDCs (red color) and the corresponding fits (black color). For the In-doped Bi_2Se_3 samples, we have taken the range of compositions that are in the topological side of the quantum-phase transition. In Supplementary Figs. 19b,d we obtain a linear dependence of the surface band gap with the linewidth broadening for both Mn and In-doped samples, respectively. This similarity strongly suggests that the mechanism underlying the surface band gap opening in both Mn and In-doped samples has the same origin, namely that the additional linewidth broadening is most likely caused by resonant processes of non-magnetic scattering for both types of impurites. This conclusion is further supported by the fact that our spin-resolved ARPES measurements shown in Supplementary Fig. 14 reveal that the out-of-plane spin polarization measured at zero momentum is zero for both gapped Dirac cones in Mn-doped (Supplementary Fig. 14a) and In-doped (Supplementary Fig. 14b) samples. The absence of a hedgehog spin texture in Mn-doped films and the similarity to the results for In-doped samples pinpoints a non-time reversal symmetry breaking mechanism underlying the origin of the surface band gap, a fact that is also consistent with our conclusions derived from the magnetic properties of the Mn-doped films.

Supplementary Note 8: Sample Growth and Structural Characterization

The In-doped bulk crystals were grown from the melt by the Bridgman method, and the growth time including cooling was about 2 weeks for a ~50 g crystal. Mn-doped Bi_2Se_3 layers were grown by molecular beam epitaxy (MBE) on cleaved (111) BaF_2 substrates, and a similar growth procedure as the one described here was employed for Sn-doped Bi_2Se_3 films. For the growth of Mn-doped Bi_2Se_3 samples, the molecular beams were generated using a compound bismuth selenide effusion cell, a Mn cell, and an additional selenium cell for stoichiometry control. A whole series of samples was grown in which the Mn concentration was varied between 0 and 8%. The MBE growth was carried out at a substrate temperature of 380°C and growth rates around 0.2 QL/sec or ~2Å/sec at a background pressure $\leq 5\times10^{-10}$ mbar. The flux rates from the individual sources were precisely measured using a water cooled quartz crystal microbalance moved into the substrate position. The resulting mass deposition rates were converted into flux rates of atoms arriving at the surface per unit area using the densities and molar masses of the different constituents. The Mn concentration x_{Mn} was determined from the obtained Mn to Bi_2Se_3 flux ratio, i.e., the ratio of arriving Mn atoms relative to the number of (Bi + Mn) atoms. The concentrations derived in this way were in good agreement with the concentrations determined by electron microprobe x-ray analysis as well as the concentrations determined from the core-level peaks in the

photoemission spectra. A high excess of Se flux above 2 Å/sec was provided for all samples, which corresponds to an overall Se-to-Bi(Mn) flux ratio of ～5:1.

As shown by the reflection high-energy electron diffraction patterns and atomic force microscopy images presented in Supplementary Fig. 20, under these conditions the growth of $(Bi_{1-x}Mn_x)_2Se_3$ proceeds in a pure 2D mode irrespective of the Mn concentration, resulting in 2D diffraction streaks normal to the surface as well as a terraced surface structure mostly exhibiting quintuple layer steps. According to our x-ray diffraction results presented in Supplementary Figs. 10 – 13, the crystal structure of the epilayers exclusively shows a *c*-axis orientation perpendicular to the surface. The bulk cubic lattice constant of BaF_2 is 6.200 Å and the in-plane lattice constant of the (111) surface 4.384 Å compared to a = 4.138 Å for Bi_2Se_3. In spite of this lattice mismatch, our layers are found to be fully relaxed from x-ray diffraction. All $(Bi_{1-x}Mn_x)_2Se_3$ epilayers exhibit n-type conduction with electron densities in the range of $1–5\times10^{19}$ cm^{-3} that only gradually increases with increasing Mn content.

Supplementary Note 9: Core-level photoemission

In Supplementary Fig. 21, we show x-ray photoemission (XPS) spectra of the Mn-doped sample with 8 % concentration. The XPS measurements were taken at an electron kinetic energy of 70 eV for (a) Mn 2*p*, (b) Se 3*d* and (c) Bi 5*d* core levels. The Mn 2*p* core level clearly contains dominant Mn^{2+} (d^5) contributions. We observe a Mn^{2+} (d^5) component (640.7 eV) with some other contributions of Mn^{+1} (639.6 eV) and Mn^{+3} (641.7eV) which are not well resolvable, plus a pronounced Mn^{2+} (d^5) satellite. Both Se 3*d* and Bi 5*d* high-resolution spectra demonstrate a multicomponent structure as compared to clean Bi_2Se_3, with two additional components being related to the chemical bonds to Mn atoms. Similar measurements were carried out for other Mn concentrations. The composition of the samples was confirmed by the XPS measurements with an accuracy of 1 mole %. The composition was quantified using atomic sensitivity factors and taking into account the calibration of the analyzer transmission function. For x=0.08 we found Mn/Bi=0.074, for x=0.04 Mn/Bi=0.044 correspondingly.

Supplementary References

[1] Shirley, D. A. High-resolution x-ray photoemission spectrum of the valence bands of gold. *Phys. Rev. B* **5**, 4709 (1972).

[2] Arrott, A. S. Equations of state along the road to Arrott's last plot. *J. Magn. Magn. Mat.* **322**, 1047-1051 (2010).

[3] Zhou, J.-S. *et al.* Critical behavior of the ferromagnetic perovskite $BaRuO_3$. *Phys. Rev. Lett.* **101**, 077206 (2008).

[4] Hor, Y. S. *et al.* Development of ferromagnetism in the doped topological insulator $Bi_{2-x}Mn_xTe_3$. *Phys. Rev. B* **81**, 195203 (2010).

[5] Bianchi, M., Hatch, R. C., Mi, J., Iversen, B. B. & Hofmann, P. Simultaneous quantization of bulk conduction and valence states through adsorption of nonmagnetic Impurities on Bi_2Se_3. *Phys. Rev. Lett.* **107**, 086802 (2011).

[6] Rosenberg, G. & Franz, M. Surface magnetic ordering in topological insulators with bulk magnetic dopants. *Phys. Rev. B* **85**, 195119 (2012).

[7] Chen, Y. L. *et al.* Massive Dirac fermion on the surface of a magnetically doped topological insulator. *Science* **329**, 659-662 (2010).

[8] Schmidt, T. M., Miwa, R. H. & Fazzio, A. Spin texture and magnetic anisotropy of Co impurities in Bi_2Se_3 topological insulators. *Phys. Rev. B* **84**, 245418 (2011).

[9] Henk, J. *et al.* Topological character and magnetism of the Dirac state in Mn-doped Bi_2Te_3. *Phys. Rev. Lett.* **109**, 076801 (2012).

[10] Xu, S.-Y. *et al.* Hedgehog spin texture and Berry's phase tuning in a magnetic topological insulator. *Nature Phys.* **8**, 616-622 (2012).

[11] Nakajima, S. The crystal structure of $Bi_2Se_{3-x}Te_x$. *J. Phys. Chem. Solids* **24**, 479-485 (1963); see also PDF-4 database (PDF-01-089-2008).

[12] H. Steiner, V. Volobuev, O. Caha, G. Bauer, G. Springholz, and V. Holy, Structure and composition of bismuth telluride topological insulators grown by molecular beam epitaxy, *J. Appl. Cryst.* **47**, 1889-1900 (2014).

[13] Hopkinson, J. F. L., Pendry, J. B. & Titterington, D. J. Calculation of photoemission spectra for surfaces of solids. *Comput. Phys. Commun.* **19**, 69-92 (1980).

[14] Braun, J. The theory of angle-resolved ultraviolet photoemission and its applications to ordered materials. *Rep. Prog. Phys.* **59**, 1267-1338 (1996).

[15] Ebert, H., Ködderitzsch, D. & Minár, J. Calculating condensed matter properties using the KKR - Green's function method - recent developments and applications. *Rep. Prog. Phys.* **74**, 096501 (2011).

[16] Braun, J., Minár, J., Matthes, F., Schneider, C. M. & Ebert, H. Theory of relativistic photoemission for correlated magnetic alloys: LSDA+DMFT study of the electronic structure of Ni_xPd_{1-x}. *Phys. Rev. B* **82**, 024411 (2010).

[17] Braun, J. *et al.* Exceptional behavior of d-like surface resonances on W(110): the one-step model in its density matrix formulation. *New J. Phys.* **16**, 015005 (2014).

[18] Black-Schaffer, A. M. & Balatsky, A. V. Strong potential impurities on the surface of a topological insulator. *Phys. Rev. B* **85**, 121103(R) (2012).

[19] Black-Schaffer, A. M. & Balatsky, A. V. Subsurface impurities and vacancies in a three-dimensional topological insulator. *Phys. Rev. B* **86**, 115433 (2012).

[20] Brahlek, M. *et al.* Topological-metal to band-insulator transition in $(Bi_{1-x}In_x)_2Se_3$ thin films. *Phys. Rev. Lett.* **109**, 186403 (2012).
